# Managing Cardiovascular Risk Factors: The Gap between Evidence and Practice

**DOI:** 10.1371/journal.pmed.0020131

**Published:** 2005-05-31

**Authors:** Fiona Turnbull

## Abstract

There are clear evidence-based guidelines for managing patients at risk of cardiovascular disease, and yet many doctors don't follow these guidelines.

Many large randomised trials have provided an abundance of high-quality evidence for the benefits of managing two major cardiovascular risk factors—high blood pressure and high cholesterol. National guidelines in turn distil this evidence and aid clinicians in making decisions about how best to manage their patients' care. However, despite the existence of clear evidence-based guidelines, many patients at risk of cardiovascular disease who should be receiving treatment are not, while many others who are on treatment are not receiving treatment in line with recommendations. A paper in last month's *PLoS Medicine* and another in this issue of the journal illustrate such “treatment gaps”.

## Two New Studies on the “Treatment Gap”

One of the papers, by Ma et al. [1], uses United States national survey data collected over a ten-year period to obtain estimates of statin use among patients categorized by their risk of coronary heart disease and presence/ absence of hyperlipidaemia. Not surprisingly, the results show a more than 2-fold increase in the proportion of patients with hyperlipidaemia treated with lipid lowering agents between 1992 and 2002— statins accounting for most of this increase. However, even at the point of highest treatment uptake, only around half of patients with hyperlipidaemia were receiving treatment.

Even more striking are the results for the use of statins in patients categorized by their cardiovascular risk. Among patients at high risk, the absolute maximum proportion of individuals receiving treatment at the end of the ten-year review period (i.e., in 2002) was only 19%. Additional analyses suggest that lower statin use in at-risk patients was associated with younger age, female gender, African-American background, and care by non-cardiologists. The authors appropriately conclude that statins remain underused —particularly among patients who have normal lipid levels but who are otherwise at high cardiovascular risk [2].

A similar evidence–practice gap, this time for blood pressure, is highlighted in the other article, by Morgan et al. [3]. In this paper, data from public, medical, hospital, and pharmaceutical programs in British Columbia are used to determine trends in the use of thiazide diuretics compared with other, more costly agents as a first-line treatment to lower blood pressure among older, newly treated patients with hypertension. The results show that only around one-third of patients received thiazide diuretics. Furthermore, even in the absence of certain comorbidities—such as diabetes, which might influence a clinician to choose an alternative agent—thiazides were used in no more than 45% of older eligible patients.

Compared with newer agents such as angiotensin receptor blockers and calcium antagonists, which cost upwards of US$1.00/day, thiazides remain the cheapest blood pressure lowering agents, costing less than $0.01/day. The authors reasonably argue that as long as thiazides remain at least equivalent to other blood pressure lowering agents in terms of reducing cardiovascular mortality and morbidity [4], their preferential use as a first-line agent can be justified on the basis of their low cost.

## Narrowing the Gap

Why do such gaps between evidence and practice exist? In 2002, around 800 primary care physicians in five European countries were surveyed to assess the acceptance and or implementation of treatment guidelines for high cholesterol and coronary heart disease (the Reassessing European Attitudes about Cardiovascular Treatment survey) [5]. Although most (89%) of those interviewed acknowledged the need for formal guidelines, and a similar proportion agreed with the content of current guidelines, only 18% of physicians believed that guidelines were being implemented to a major extent, indicating a problem with either their understanding or implementation. The barriers to implementation that were most commonly cited by physicians in the survey are shown in Table 1.

**Table 1:**
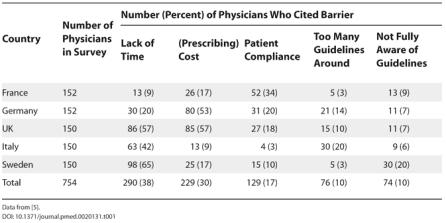
The Reassessing European Attitudes about Cardiovascular Treatment Survey: Most Commonly Cited Barriers to Implementation of Coronary Heart Disease Guidelines

Data from [5]

Perhaps the two most important means by which improved use of treatment guidelines can be achieved are (1) improving the understanding of the basic concepts that underpin them and (2) reducing the number and complexity of the main messages. In terms of addressing the first of these, an understanding of the concept of “absolute risk”—the probability of a patient developing a cardiovascular event over a specified time period—is crucial.

An absolute risk approach to cardiovascular prevention acknowledges that the presence of small or moderate elevations of multiple risk factors often confer greater risk of cardiovascular disease than an extreme elevation of a single risk factor. Furthermore, the nature of the association between blood pressure, cholesterol, and cardiovascular disease implies that a given reduction in the level of the risk factor, regardless of baseline level, will reduce cardiovascular risk by a constant proportion. Therefore, the goal of blood pressure lowering and lipid lowering is not to “normalize” levels but to reduce them as much as possible, and this means targeting everyone at high risk as determined by age or known cardiovascular disease rather than by the level of the risk factors [6]. This approach (“the lower, the better”) to both blood pressure and cholesterol management in high-risk individuals has been supported by recent meta-analyses and large trials [7,8].

Adopting an absolute-risk-based approach requires a paradigm shift and challenges the way doctors have traditionally made treatment decisions based on single risk factor levels. Although there is some evidence that clinical practice is conforming to the notion of risk stratification, other studies suggest that a large proportion of clinicians, particularly those in primary care, still do not use risk charts [5]. While it is acknowledged that the absolute risk approach has some limitations, integration of this approach into clinical care will be the key to future major gains in the prevention of cardiovascular disease.
